# Associations between Green Building Design Strategies and Community Health Resilience to Extreme Heat Events: A Systematic Review of the Evidence

**DOI:** 10.3390/ijerph16040663

**Published:** 2019-02-24

**Authors:** Adele Houghton, Carlos Castillo-Salgado

**Affiliations:** 1 Biositu, LLC, Houston, TX 77006, USA; 2 Department of Epidemiology, Johns Hopkins Bloomberg School of Public Health, Baltimore, MD 21205, USA; ccastil3@jhu.edu

**Keywords:** heat-related hazards, sustainable design, climate change mitigation, climate change adaptation, sustainable communities

## Abstract

This project examined evidence linking green building design strategies with the potential to enhance community resilience to extreme heat events. Following the Preferred Reporting Items for Systematic Reviews and Meta-Analyses (PRISMA) method for a systematic review, it assessed the strength of the evidence supporting the potential for Leadership in Energy and Environmental Design (LEED®) credit requirements to reduce the adverse effects of extreme heat events and/or enhance a building’s passive survivability (i.e., the ability to continue to function during utility outages) during those events. The PRISMA Flow Diagram resulted in the selection of 12 LEED for New Construction (LEED NC) credits for inclusion in the review. Following a preliminary scan of evidence supporting public health co-benefits of the LEED for Neighborhood Development rating system, queries were submitted in PubMed using National Library of Medicine Medical Subject Headings Terms. Queries identified links between LEED credit requirements and risk of exposure to extreme heat, environmental determinants of health, co-benefits to public health outcomes, and co-benefits to built environment outcomes. Public health co-benefits included reducing the risk of vulnerability to heat stress and reducing heat-related morbidity and mortality. The results lay the groundwork for collaboration across the public health, civil society, climate change, and green building sectors.

## 1. Introduction

The health effects of climate change are significant and growing [1]. Direct effects include heat stress; injuries from flooding; and, intensification of chronic conditions such as cardiovascular disease [2,3,4], respiratory disease [2,3,4], and hypertension [3,4]. Indirect health effects include waterborne diseases [4,5], food insecurity [4,5], increased incidence of vector-borne infectious diseases (particularly in regions with historically low exposure rates) [4,5,6], and increased seasonal allergies [4,5]—all of which are exacerbated by rising temperatures [7]. Heat waves alone are directly responsible for between 670 [8] and 1300 [9] deaths per year in the U.S., depending on the calculation methodology. As temperatures continue to warm, climate projections estimate the number of premature deaths directly or indirectly associated with extreme heat to rise to the thousands or even tens of thousands by the end of the century [10,11,12,13,14,15,16,17].

The built environment is a major contributor to both the cause of climate change (i.e., greenhouse gas (GHG) emissions) and the extent of a community’s resilience (i.e., its ability to respond adequately to changing climatic conditions) [18] to its effects, such as more frequent and more intense heat waves. The research presented in this article seeks to identify green building design strategies that fall within the definition of community resilience outlined in the 2016 report published by the U.S. Global Change Research Program, *The Impacts of Climate Change on Human Health in the United States*: “… the ability to prepare and plan for, absorb, recover from, and more successfully adapt to adverse events” [7], (p. 30). According to this definition, community resilience encompasses both interventions that reduce the risk of injury, disease, and death, and strategies that avoid disruptions to the built environment (such as the failure of building systems, a building’s envelope, or its structural systems). For extreme heat events, community resilience refers to policies and interventions that: reduce the risk of population and/or built environment exposure to extreme heat, minimize environmental determinants of health leading to exposure, and have the potential to lead to co-benefits to public health and/or built environment outcomes.

According to the U.N. Environment Program, the building sector is estimated to contribute up to 30% of anthropogenic (or human-caused) GHG emissions globally, primarily through energy use during building operations [19]. Land use policies can also exacerbate underlying social and health vulnerabilities. For example, during heat waves, ambient air temperatures have been found to be higher in low income urban neighborhoods (characterized by large swathes of dark, impervious surfaces) than in high income neighborhoods in the same municipality (which are often characterized by tree canopies and other forms of vegetation [20]).

U.S. Global Change Research Program has identified buildings and structures as a focus for resilience efforts; because, when they fail, both the human health and economic ramifications can be far-reaching. The U.S. Climate Resilience Toolkit identifies “climate-smart building” as an approach to design and development that takes into account the dangers of climatic events such as extreme heat in an effort to reduce the risk of failure during and after extreme weather events [21]. Many of the measures promoted by environmentally conscious, or “green,” building practices have the potential protect building occupants and the surrounding community from the negative health and economic effects of climatic events. The question is which design practices are protective during heat waves and in what way.

### 1.1. Green Building Practices and Population Health

Green building best practice guides have proliferated globally over the past 20 years. U.S. Green Building Council’s (USGBC) Leadership in Energy and Environmental Design® (or, LEED) family of rating systems, which is based in the U.S., is generally recognized as the market leader worldwide. Other best practice guides include BREEAM (https://www.breeam.com/) based in the U.K., CASBEE (http://www.ibec.or.jp/CASBEE/english/) based in Japan, Green Star (https://new.gbca.org.au/green-star/) based in Australia, Living Building Challenge (https://living-future.org/lbc/) based in the U.S. and Canada, and a number of products specializing in specific market segments – such as Enterprise Green Communities (https://www.enterprisecommunity.org/solutions-and-innovation/green-communities/criteria) for affordable housing, RELi (http://c3livingdesign.org/?page_id=13783) for resilient design, and SITES (http://www.sustainablesites.org/) for land development and management. Two new rating systems, Fitwel (https://fitwel.org/) and WELL (https://www.wellcertified.com/), were recently launched with a focus on optimizing occupant health and wellbeing.

The systematic review presented in this article provided the underlying research for a spatial analysis project designed to assess the strength of association between the green building credits awarded to LEED for New Construction and Major Renovation (LEED NC) certified projects in two U.S. cities in the first decade of the 21st century and the location of neighborhoods that had been identified by vulnerability indices to be particularly at risk of negative health outcomes after exposure to extreme heat events. Because the spatial analysis was focused on the U.S., the researchers searched for LEED certified projects and therefore designed the systematic review around LEED, which is the dominant green building rating system in the U.S. LEED NC was selected for review over LEED for Neighborhood Development (LEED ND, which focuses on the neighborhood scale, rather than the building scale) for two reasons: (1) The number of LEED ND projects in the study area was too small to conduct a spatial analysis based on that rating system, and (2) the vast majority of green building practices outlined in other green building best practice systems (including LEED ND) share fundamental concepts with the prerequisites and credits in LEED NC. As a result, the review used LEED NC credits and the body of evidence that was available to project teams for the decade from 2002 to 2012 to generate a broad understanding of the state of the evidence linking green building strategies to community resilience in the face of extreme heat events.

LEED is an internationally recognized, third party-verified family of green building rating systems that takes a multi-attribute approach to defining an environmentally sensitive building or neighborhood. While it is based in the U.S., LEED is in wide use globally. It covers a large swath of real estate activity, including residential construction, commercial construction, existing buildings, and neighborhood development projects. This review focused on the 2009 version of the flagship rating system, LEED for New Construction and Major Renovations, which is divided into five categories equaling 110 possible points: Sustainable Sites, Water Efficiency, Energy and Atmosphere, Indoor Environmental Quality, and Innovation in Design. The rating system combines mandatory “prerequisites,” which set a baseline of environmental performance for all certifying projects, with voluntary “credits,” which allow project teams to tailor LEED compliance with the unique attributes of a specific project. Most prerequisites and credits are performance-based rather than prescriptive. That is, they set a measurable goal (such as reducing the heat island effect) but do not mandate how the project achieves the minimum threshold. Notably, it is not possible to achieve every credit in the LEED rating system. The framework was developed with an eye to providing a pathway to achievement for many types of projects—large and small, urban and rural. As a result, the achievement of some credits may preclude achievement of other credits—such as a project located in a dense urban area achieving Sustainable Sites Credit 2: Development Density and Community Connectivity but finding it prohibitively expensive to achieve Sustainable Sites Credit 5.1: Site Development—Protect or Restore Habitat or Sustainable Sites Credit 5.2: Site Development—Maximize Open Space (see Table 1 for credit details). Since some credit-compliant strategies are more likely than others to benefit public health, it is most appropriate to interpret achievement of a LEED prerequisite or credit as demonstrating the *potential* for a building project to enhance community health resilience to a climatic event such as extreme heat, rather than as direct evidence of resilience.

### 1.2. Green Building Practices and Climate Change

LEED for New Construction and Major Renovations devotes close to half of all available points to reducing GHG emissions, mainly through energy efficiency measures, on-site renewable energy installations, and reducing single-occupancy vehicle use [22]. While adaptation to climate change is not specifically identified as a priority for the LEED rating systems, two studies have found associations between LEED credits and built environment resilience. Larsen [23] outlines “No Regrets” and “Resilient” strategies linked with LEED credits that have the potential to protect both a building’s structure and its ability to operate following a climatic event. A subsequent study sponsored by the USGBC [24] compiled indices that assign relative values to LEED credits based on: (a) their level of reliance on assumptions that may shift as a result of climate change or (b) their potential to enhance a building’s adaptive capacity after exposure to climatic events. Neither study addresses population vulnerability to climatic events, particularly in relation to the localized vulnerability of a project site to the health and environmental effects of climatic events.

This article assesses the strength of the evidence supporting the potential for LEED credit requirements to reduce the adverse effects of extreme heat and/or enhance a building’s passive survivability (i.e., the ability to continue to function during utility outages [25]) during those events. By identifying the health co-benefits of certain green building strategies, this systematic review seeks to demonstrate the evidence of potential for cross-sectorial collaboration among public health departments and their partners in the real estate, planning, emergency, and transportation sectors around community resilience to extreme heat.

## 2. Materials and Methods

### 2.1. Systematic Review

A systematic review is defined by the Preferred Reporting Items for Systematic reviews and Meta-Analyses (PRISMA) method as “a review of a clearly formulated question that use systematic and explicit methods to identify, select, and critically appraise relevant research, and to collect and analyze data from the studies that are included in the review. Statistical methods (meta-analysis) may or may not be used to analyze and summarize the results of the included studies” [26]. Given the small body of research and exploratory nature of studies exploring the links between green building design strategies and environmental exposure to extreme heat and community resilience to extreme heat events, we performed a systematic review without an accompanying meta-analysis.

### 2.2. Conceptual Framework

**Figure 1:**
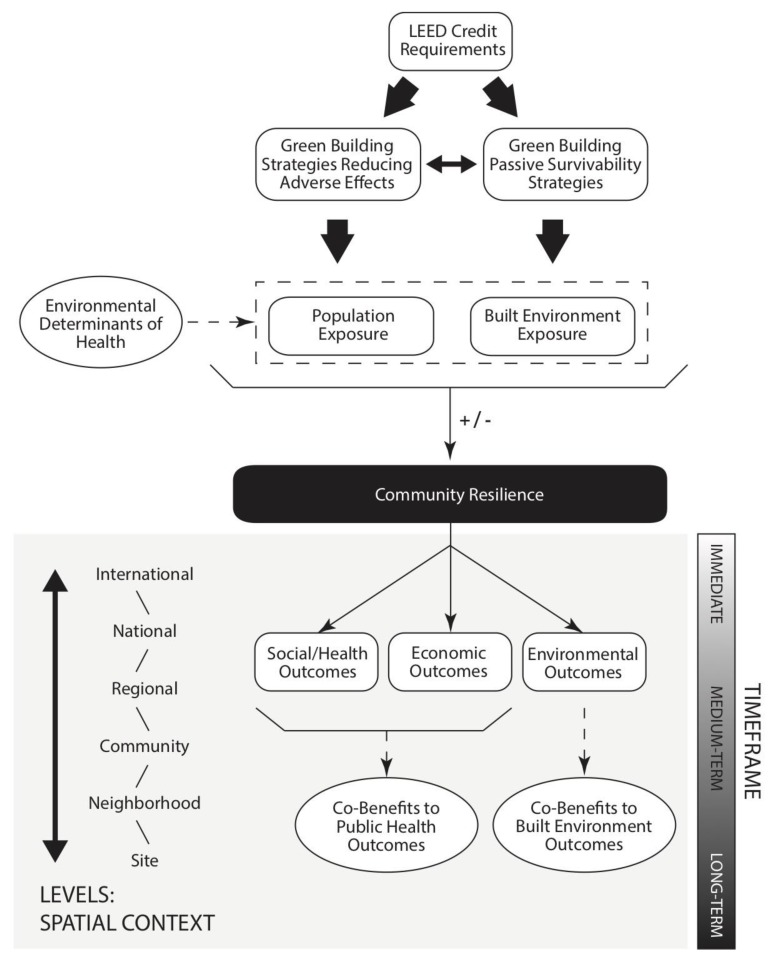
Conceptual framework: establishing an evidence base for associations between Leadership in Energy and Environmental Design (LEED®) credit requirements and climate change resilience outcomes.

The conceptual framework for the systematic review (Figure 1) adapts the social determinants of health conceptual framework [27,28,29] to establish a pathway for linking LEED with public health outcomes associated with climate change. Because LEED credit requirements address many aspects of building design and operations, they can result in outcomes affecting a number of spatial scales, from the building site to the neighborhood, community, regional, national, or even international scale [27]. The timeframe between the strategy’s implementation and an associated health outcome can likewise range from immediate to the short-term or long-term future [27].

The co-benefits resulting from the green building design process can directly impact two of the major environmental determinants of health associated with climatic events: population risk of exposure and built environment risk of exposure. The combination of these determinants influences a community’s relative resilience to specific climatic events, such as extreme heat. After exposure to a climatic event, the strength of community resilience can be measured through three outcomes: social/health outcomes, economic outcomes, and environmental outcomes. Therefore, if a LEED credit is implemented with its potential impact on community resilience in mind, its implementation can result in co-benefits to public health outcomes and/or built environment outcomes. It is important to reiterate that completion of a LEED credit requirement only indicates the potential for co-benefits to public health and the built environment. The magnitude of that co-benefit will depend on the type and level of environmental exposure, the building’s function, the characteristics of vulnerable populations on-site and in the surrounding neighborhood, and complementary activities that may bolster resilience but not improve a project’s final LEED point tally.

### 2.3. LEED Credit Inclusion Criteria

**Figure 2:**
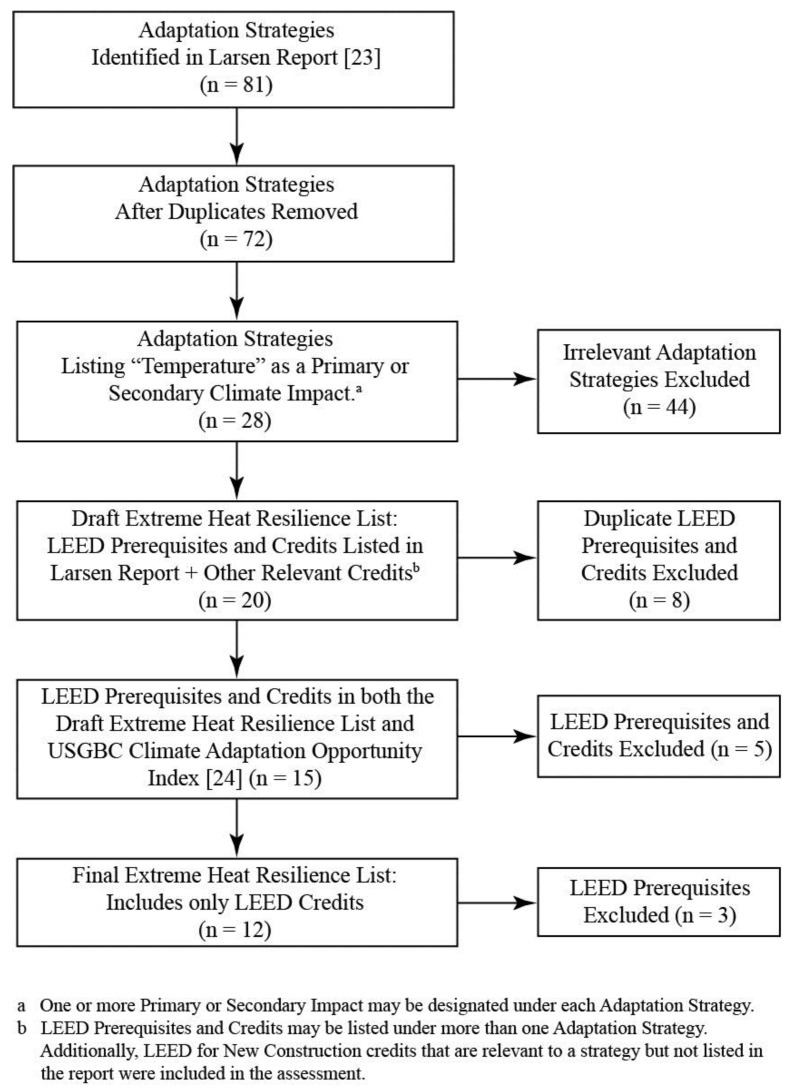
Flow chart of LEED Credit inclusion criteria. Diagram adapted from the Preferred Reporting Items for Systematic Reviews and Meta-Analyses (PRISMA) Flow Diagram [26].

**Figure A1:**
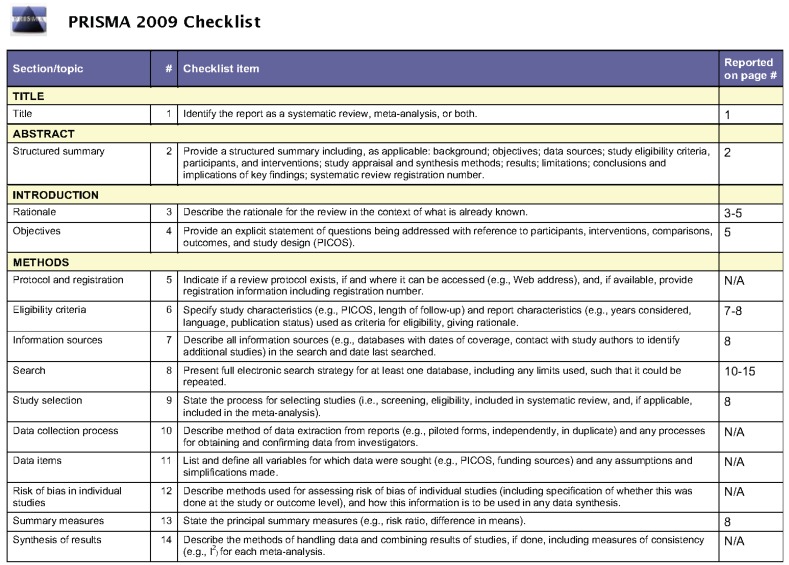

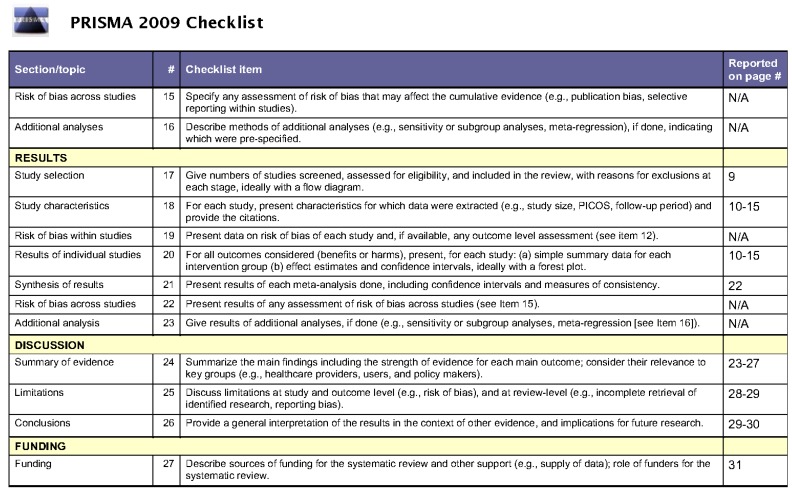
PRISMA Checklist. Source: Moher, D.; Liberati, A.; Tetzlaff, J.; Altman, D.G.; Altman, D.; Antes, G.; Atkins, D.; Barbour, V.; Barrowman, N.; Berlin, J.A.; et al. Preferred reporting items for systematic reviews and meta-analyses: The PRISMA statement [26].

Following PRISMA guidelines (Figure 2, Figure A1), the review began with an assessment of the LEED credits referenced by 81 adaptation strategies associated with green building design outlined by Larsen [23]. Each adaptation strategy identified LEED credits with the potential to enhance a project’s ability to adapt to one of a suite of climate change-related hazards. Adaptation strategies were included in the systematic review analysis if “Temperature” was identified as either a primary or secondary climate impact in Larsen et al. (28 strategies). Examples include: shading devices, high performance glazing, roof and wall insulation, white and vegetated roofs, compact and mixed-use development, and increased vegetation. LEED prerequisites and credits that were identified as linked to an adaptation strategy were then included in the draft Extreme Heat Resilience list. As some prerequisites and credits were listed under more than one adaptation strategy in Larsen et al., duplications were removed. Credits that were relevant to an adaptation strategy but not included in the Larsen report (such as the ability of Energy & Atmosphere Credit 2: On-Site Renewable Energy to potentially enhance a building’s passive survivability during a heat-related power outage) were then added to the list, resulting in 20 Prerequisites and Credits. The draft list of LEED credits was then cross-referenced with the Climate Adaptation Opportunity Index developed by Pyke [24]. Five prerequisites and credits that did not appear in Pyke were excluded. And, finally, two prerequisites were removed from the analysis, because they are required for all LEED certified projects. In the end, 12 credits were included in the Extreme Heat Resilience systematic review (Table 1).

### 2.4. Systematic Review Inclusion Criteria

**Figure A2:**
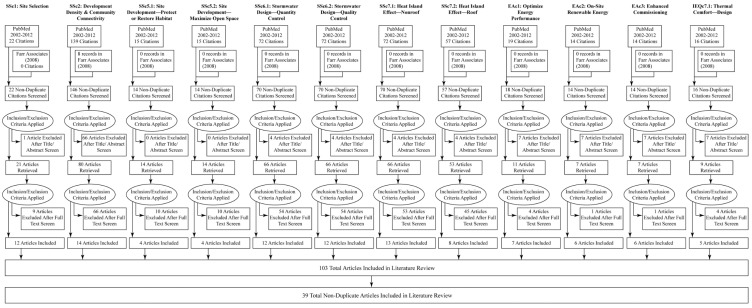
Flow chart of extreme heat systematic review article inclusion criteria. Source: Moher, D.; Liberati, A.; Tetzlaff, J.; Altman, D.G.; Altman, D.; Antes, G.; Atkins, D.; Barbour, V.; Barrowman, N.; Berlin, J.A.; et al. Preferred reporting items for systematic reviews and meta-analyses: The PRISMA statement [26].

The systematic review (Appendix A) began by confirming whether or not the LEED credits in the Extreme Heat Resilience list were also addressed by Farr [30], a review of the evidence supporting public health co-benefits of the LEED for Neighborhood Development (LEED ND) rating system that was commissioned by the USGBC. No similar review has been published identifying public health evidence supporting specific credits in the LEED for New Construction (LEED NC) rating system—the most widely used version of LEED. However, the substance of many LEED ND credits is duplicated at the site scale in LEED NC. The systematic review was therefore supplemented with relevant references in the Farr report (Figure A2).

**Figure 3:**
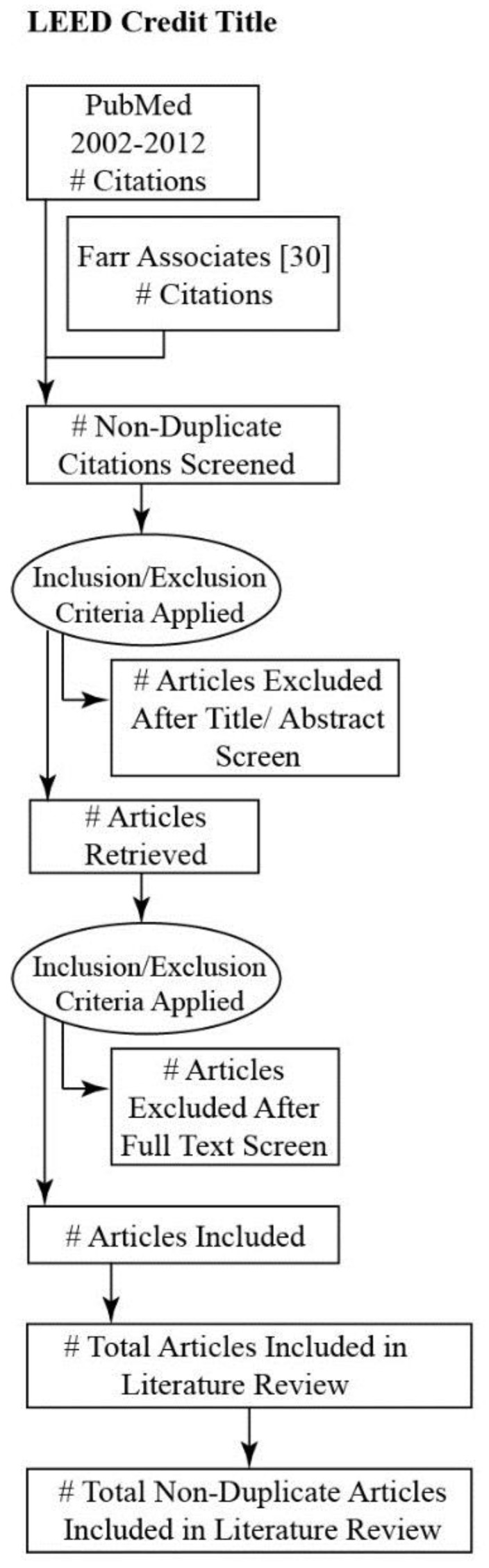
Flow chart of generic article inclusion criteria. Diagram adapted from PRISMA Flow Diagram [26].

Following this preliminary review, queries were submitted in PubMed using National Library of Medicine Medical Subject Headings (MeSH®) [31] Terms. In some cases, more than one set of MeSH terms was submitted for a single credit. For example, for Sustainable Sites Credit 2: Development Density and Community Connectivity, the following MeSH queries were submitted. Related to Option 1—Development Density, the MeSH terms were: “Population Density” AND “Climate Change” AND “Urban Health”. Related to Option 2—Community Connectivity, the MeSH terms were: “Population Density” AND “Urban Health”. Additionally, eight citations were reviewed from the Farr report, five of which were relevant to the enquiry [32,33,34,35,36]. For simplicity’s sake, MeSH terms have been listed in Table A1 without outlining specific combinations. The Table A1 entry for Sustainable Sites Credit 2 lists “Climate Change,” “Population Density,” and “Urban Health” without reference to query combinations. See Appendix Table A2 for a table outlining the query combinations for specific LEED credits. Duplicate citations were removed. And, only articles in English from 2002 to 2012 were included in the analysis. Titles and abstracts were reviewed for evidence of links between strategies that could be used to comply with LEED credit requirements and their potential impact on the major elements of the conceptual framework (Figure A2): risk of exposure to extreme heat, environmental determinants of health (i.e., population exposure and/or built environment exposure), co-benefits to public health outcomes (i.e., relevant social/health outcomes, economic outcomes, and/or environmental outcomes), and co-benefits to built environment outcomes (i.e., relevant environmental outcomes). Articles that addressed one or more of these criteria were selected for a full text review. See Figure 3 for a generic flow chart illustrating the article inclusion process in general and Figure A2 for details on LEED credit included in the review.

## 3. Results

### 3.1. LEED Credits Included in the Review

Of the 12 LEED credits included in the Extreme Heat Resilience systematic review (Table 1), eight were drawn from the Sustainable Sites category (Sustainable Sites Credit 1: Site Selection, Sustainable Sites Credit 2: Development Density and Community Connectivity, Sustainable Sites Credit 5.1: Site Development—Protect or Restore Habitat, Sustainable Sites Credit 5.2: Site Development—Maximize Open Space, Sustainable Sites Credit 6.1: Stormwater Design—Quantity Control, Sustainable Sites Credit 6.2: Stormwater Design—Quality Control, Sustainable Sites Credit 7.1: Heat Island Effect—Nonroof, Sustainable Sites Credit 7.2: Heat Island Effect—Roof), three were drawn from the Energy & Atmosphere category (Energy and Atmosphere Credit 1: Optimize Energy Performance, Energy and Atmosphere Credit 2: On-Site Renewable Energy, Energy and Atmosphere Credit 3: Enhanced Commissioning), and one was drawn from the Indoor Environmental Quality category (Indoor Environmental Quality Credit 7.1: Thermal Comfort—Design).

A thirteenth credit (Indoor Environmental Quality Credit 7.2: Thermal Comfort—Verification) was removed from the final list due to insufficient evidence of positive co-benefits to public health and built environment outcomes. Table A3 provides a crosswalk of these LEED credits in comparison with other green building best practice standards around the world.

### 3.2. Results by LEED Credit

The initial query returned 535 articles. After applying the systematic review inclusion criteria, 103 total articles were included in the full text review, 39 of which were non-duplicative (Figure A2). However, it should be noted that multiple reviews of a single article do not necessarily indicate duplicative results, because each review assessed potential strategies for achieving a specific LEED Credit. Likewise, all co-benefits and co-harms in Table 2 and Table 3 (and displayed in more detail in Table A1 in the Appendix A) are contingent upon the population and environmental conditions specific to a project site.

#### 3.2.1. Sustainable Sites Credit 1: Site Selection

This credit requires projects to avoid development in or adjacent to prime farmland, endangered species habitat, parkland, floodplains, wetlands, and water bodies (Table A1). A review was conducted for the first three topic areas: prime farmland, endangered species habitat, and parkland. The final three land types were removed from the systematic review, because they are not directly applicable to heat resilience.

Prime farmland queried “Agriculture,” “Climate Change,” “Facility Design and Construction,” and “Urbanization.” (17 citations were returned, six of which were relevant to the inquiry [18,37,38,39,40,41].) Avoiding development in these areas was found to reduce the risk of exposure to extreme heat events by not contributing to sprawl [18,37,38,39], impervious cover [40], or increasing the extent of the urban heat island effect [41]. By positively affecting the associated environmental determinants of health (air pollution [18], access to opportunities to exercise [18], dependence on automobiles [18], food and nutrition security [37,38], food safety [37,38], and habitat fragmentation [39,40]), these practices were found to reduce the risk of heat-related and cardiovascular morbidity and mortality [18,41], under- and mal-nutrition [37,38], infectious disease [39], and interaction between wildlife and humans [39]. The co-benefits to built environment outcomes were identified as mitigating the urban heat island effect [18]; reducing development in areas without services [18]; and, increasing access to local, productive agricultural land [37,38].

Endangered species habitat queried “Biodiversity” and “Urban Health.” (seven citations were returned, four of which were relevant to the inquiry [42,43,44,45].) Avoiding development in these areas was found to reduce the risk of exposure to extreme heat events by not contributing to sprawl [44,45] or the urban heat island effect [42,43]. By positively affecting the associated environmental determinants of health (air pollution [44], biodiversity in urban environments [42,43], and disease-carrying vectors [45]), these practices were found to improve mental health and wellbeing [42] and reduce the risk of respiratory disease [44] and vector-borne diseases [45]. More recent studies expand the list of vector-borne diseases covered by the systematic review to include the recent outbreak of the Zika virus in the Americas [6,46]. The co-benefits to built environment outcomes were identified as mitigating the urban heat island effect [44], increasing native vegetation and pervious surface [42,43], increasing street trees [42,44], and reducing ground-level ozone pollution [44].

Parkland queried “Biodiversity,” “Conservation of Natural Resources,” and “Facility Design and Construction.” (four citations were returned, two of which were relevant to the inquiry [47,48].) Avoiding development in these areas was found to reduce the risk of exposure to extreme heat events by not contributing to sprawl [47,48]. By positively affecting the associated environmental determinant of health (habitat fragmentation [47]), this practice was found to reduce the risk of interaction between wildlife and humans [47,48]. The co-benefits to built environment outcomes were identified as mitigating the urban heat island effect [47,48], encouraging clustered development [47], and increasing native vegetation [47] and pervious surface [47,48].

#### 3.2.2. Sustainable Sites Credit 2: Development Density and Community Connectivity

This credit requires projects to locate buildings on urban infill sites or on previously developed sites that are located near a residential neighborhood and basic services (such as grocery stores and banks) (Table A1). The systematic review queried “Climate Change,” “Population Density,” and “Urban Health.” One hundred and forty seven citations were returned, 14 of which were relevant to the inquiry [32,33,34,35,36,49,50,51,52,53,54,55,56,57]. Locating development in these areas was found to reduce the risk of exposure to extreme heat events by not increasing the extent of the urban heat island effect [32,33,34,35,36,49,50,51,52,53,54,55,56,57]. By positively affecting the associated environmental determinants of health (urban density [33,54], population density [33,50,55] street connectivity [33], access to multiple services [32,33,34,35,36,51,52,53], and walkability [32,34,35,36,51,52,53,55,56], these practices were found to reduce the risk of cardiovascular disease [32,33,34,35,36,50,51,52,53,55,56] (which can be exacerbated during heat events) because they encourage increased physical activity [32,33,34,35,36,51,52,53,56]. They were also found to reduce heat stress via small-scale, targeted interventions [54]. And, they create the conditions for community members to check on socially isolated neighbors [49]. However, locating a project in urban infill could also increase the risk of heat-related morbidity and mortality [49], cardiovascular disease [49] and respiratory disease [49] due to increased exposure to the urban heat island effect. The co-benefits to built environment outcomes were identified as mitigating the regional urban heat island effect through reducing sprawl development [57]; increasing access to mass transit [49]; and, performing small-scale improvements to infrastructure that benefit a large number of people [54]. An unintentional negative outcome to the built environment is the risk of increased microclimate temperatures [49], unless mitigating practices such as vegetative cover and white roofs are included in the design.

#### 3.2.3. Sustainable Sites Credit 5.1 and Sustainable Sites Credit 5.2

The results of the systematic review were identical for Sustainable Sites Credit 5.1 and Sustainable Sites Credit 5.2 (Table A1). *Sustainable Sites Credit 5.1: Site Development—Protect or Restore Habitat* requires projects to limit habitat disturbance during construction or to restore habitat. *Sustainable Sites Credit 5.2: Site Development—Maximize Open Space* requires projects to increase vegetated open space. The systematic review queried “Climate Change,” “Extreme Heat,” and “Environment Design.” Fifteen citations were returned, four of which were relevant to the inquiry [3,58,59,60]. Strategies meeting the credit requirements were found to reduce the risk of exposure to extreme heat events by reducing the urban heat island effect [3,58,59,60]. By positively affecting the associated environmental determinants of health (percentage vegetation in neighborhoods with vulnerable populations [3,58,59,60]), these practices were found to reduce vulnerability to heat stress [3,58,59] and to assist local officials in targeting early heat warning systems to neighborhoods with a combination of high land surface temperature and vulnerable populations [60]. The co-benefits to built environment outcomes were identified as improving the thermal comfort of the site and neighborhood microclimate [59,60] and reducing the burden on air conditioning systems [3].

#### 3.2.4. Sustainable Sites Credit 6.1 and Sustainable Sites Credit 6.2

The results of the systematic review were identical for Sustainable Sites Credit 6.1 and Sustainable Sites Credit 6.2. The results for Sustainable Sites Credit 7.1 added one additional reference [61], but returned the same substantive results as the previous two credits (Table A1). *Sustainable Sites Credit 6.1: Stormwater Design—Quantity Control* requires projects to design the site to reduce post-development peak discharge quantities of stormwater after heavy precipitation events. *Sustainable Sites Credit 6.2: Stormwater Design—Quality Control* requires projects to design the site to remove pollution from stormwater runoff. *Sustainable Sites Credit 7.1: Heat Island Effect—Nonroof* requires projects to shade impervious surfaces on-site or install light-colored or pervious surfaces. The systematic review queried “Climate Change,” “Environment Design,” “Extreme Heat,” and “Urbanization.” Seventy two citations were returned, 13 of which were relevant to the inquiry [3,41,58,59,60,61,62,63,64,65,66,67,68]. Strategies meeting the credit requirements were found to reduce the risk of exposure to extreme heat events by not contributing to sprawl [65] and by reducing the urban heat island effect [41,62,63,65,66,67,68]. By positively affecting the associated environmental determinant of health (percentage vegetation in neighborhoods with vulnerable populations [3,41,58,59,60,61,62,63,64,65,66,67,68], these practices were found to reduce vulnerability to heat stress [3,58,59,62,63,64,65] and to assist local officials in targeting early heat warning systems to neighborhoods with a combination of high land surface temperature and vulnerable populations [60]. A potential negative health outcome was identified as increasing pollen-producing plants [61], which could increase the risk of respiratory disease. The co-benefits to built environment outcomes were identified as improving the thermal comfort of the site and neighborhood microclimate [41,59,60,62,63,65,66,67,68], reducing the burden on air conditioning systems [3,62], and reducing localized air pollution [62].

#### 3.2.5. Sustainable Sites Credit 7.2: Heat Island Effect—Roof

This credit requires projects to install light colored and / or vegetated roofs on their buildings (Table A1). The systematic review queried “Climate Change” and “Urbanization.” Fifty seven citations were returned, eight of which were relevant to the inquiry [3,41,62,63,64,65,66,67]. Strategies meeting the credit requirements were found to reduce the risk of exposure to extreme heat events by reducing the urban heat island effect [3,41,62,63,64,65,66,67]. By positively affecting the associated environmental determinant of health (exposure to high temperatures in urban areas [3,41,62,63,64,65,66,67]), these practices were found to reduce vulnerability to heat stress [3,62,63,64] and to assist local officials in targeting early heat warning systems to neighborhoods with a combination of high land surface temperature and vulnerable populations [3]. The co-benefits to built environment outcomes were identified as improving the thermal comfort of the site and neighborhood microclimate [3,41,62,63,65,66,67], reducing the burden on air conditioning systems [3,62], reducing localized air pollution [62], and preserving green space [3,63,64].

#### 3.2.6. Energy and Atmosphere Credit 1: Optimize Energy Performance

This credit requires projects to reduce energy use in buildings and / or increase the use of on-site renewable power (Table A1). The systematic review queried “Conservation of Energy Resources,” “Cities,” “Climate Change,” “Disasters,” “Electricity,” “Environment Design,” “Facility Design and Construction,” and “Urban Health.” Nineteen citations were returned, seven of which were relevant to the inquiry [18,69,70,71,72,73,74]. Strategies meeting the credit requirements were found to reduce the risk of exposure to extreme heat events by not contributing to the urban heat island effect [18,69] and by reducing the risk of power outages caused by peak demand during heat events [69,70,71,72,73,74]. By positively affecting the associated environmental determinants of health (exposure to high temperatures in urban areas [69,71,72] and power outages exacerbated by heat [69,70,71,72,73,74]), these practices were found to reduce exposure to heat [18,69,71], poor air quality [71], exertion [71], and psychological stress during a power outage [71]. They can also assist local officials in prioritizing public health interventions in areas with high percentages of vulnerable populations (i.e., elderly, patients dependent on electrically powered medical devices, etc.) [70,71,73,74]. The co-benefits to built environment outcomes were identified as increasing the effectiveness of passive survivability (e.g., the ability of a building to operate during utility outages) [69,70,71,72,73,74], reducing the heat generated outdoors by air conditioning systems [69], increasing the thermal protection of occupants during events [69], reducing a building’s burden on the electrical grid [69], and reducing the generation of localized air pollution [71].

#### 3.2.7. Energy and Atmosphere Credit 2 and Energy and Atmosphere Credit 3

The results of the systematic review were identical for Energy and Atmosphere Credit 2 and Energy and Atmosphere Credit 3 (Table A1). *Energy and Atmosphere Credit 2: On-Site Renewable Energy* requires projects to use on-site renewable energy systems. *Energy and Atmosphere Credit 3: Enhanced Commissioning* requires projects to verify that the building’s energy systems perform as efficiently as designed. The systematic review queried “Conservation of Energy Resources,” “Cities,” “Climate Change,” “Disasters,” “Electricity,” “Urban Health.” Fourteen citations were returned, six of which were relevant to the inquiry [69,70,71,72,73,74]. Strategies meeting the credit requirements were found to reduce the risk of exposure to extreme heat events by reducing the risk of power outages caused by peak demand during heat events [69,70,71,72,73,74]. By positively affecting the associated environmental determinants of health (exposure to high temperatures in urban areas [69,71,72] and power outages exacerbated by heat [69,70,71,72,73,74]), these practices were found to reduce exposure to heat [69,71], poor air quality [71], exertion [71], and psychological stress during a power outage [71]. They can also assist local officials in prioritizing public health interventions in areas with high percentages of vulnerable populations [70,71,73,74], because low income populations are simultaneously more vulnerable to the negative health effects of extreme heat and more likely to inhabit buildings that experience power outages when the grid is overwhelmed during heat events. The co-benefits to built environment outcomes were identified as increasing the effectiveness of passive survivability [69,70,71,72,73,74], reducing the heat generated outdoors by air conditioning systems [69], increasing the thermal protection of occupants during events [69], reducing a building’s burden on the electrical grid [69], and reducing the generation of localized air pollution [71].

#### 3.2.8. Indoor Environmental Quality Credit 7.1: Thermal Comfort—Design

This credit requires projects to balance air temperature, humidity, and air speed to provide a space that is comfortable to occupants (Table A1). The systematic review queried “Conservation of Energy Resources,” “Cities,” “Climate Change,” “Disasters,” “Electricity,” “Environment Design,” “Facility Design and Construction, “Urban Health.” Sixteen citations were returned, five of which were relevant to the inquiry [18,69,70,71,72]. Strategies meeting the credit requirements were found to reduce the risk of exposure to extreme heat events by reducing the urban heat island effect [18,69] and reducing the risk of power outages caused by peak demand during heat events [69,70,71,72]. By positively affecting the associated environmental determinants of health (exposure to high temperatures in urban areas [18,70,71], power outages exacerbated by extreme heat events [69,70,71], and ventilation design [18,69]), these practices were found to improve indoor air quality [69] and reduce the risk of: mold growth [69], heat-related health effects [18,70,71,72], foodborne disease [72], and increases in rodent populations [72]. They were also found to reduce exposure to heat [18,70,71], poor air quality [18,69,71], exertion [71], and psychological stress during a power outage [71]. The co-benefits to built environment outcomes were identified as increasing the effectiveness of passive survivability [70,71,72] and protecting indoor air quality during extreme weather events [18,69].

## 4. Discussion

The systematic review revealed a number of commonalities that could be leveraged to maximize a green building’s protective features associated with extreme heat. The most frequently identified environmental determinants of health were: (1) the percentage of vegetation in neighborhoods with vulnerable populations; and, (2) exposure to high temperatures in urban areas. Power outages exacerbated by heat were also called out for the credits in the Energy and Atmosphere and Indoor Environmental Quality categories. Most of the LEED credits in the review reduced risk of exposure by reducing the urban heat island effect, a practice that was also the most frequent co-benefit to the built environment. The other two co-benefits to the built environment resulting from green building practices were: reducing the burden placed on the building’s air conditioning system and reducing the burden placed on the municipal electrical grid. Co-benefits to health included: reducing population vulnerability to heat stress, reducing heat-related injuries and mortalities, and increasing passive survivability. Similar to Larsen et al. [23], the review surfaced several themes that repeated across credits, because LEED requirements offer a variety of pathways to achieving similar goals associated with enhancing resilience to extreme heat events. The urban heat island was most often identified in the studies with increased risk of heat-related morbidity and mortality and risk of cardiopulmonary morbidity and mortality (Table 2). Mental health and social cohesion, on the other hand, were most closely associated with the Energy and Atmosphere credits, which focus on reducing the burden on the electrical grid and enhancing passive survivability during power outages (Table 2). The design opportunities (Table 3) associated with each credit emphasized the need to combine increased vegetation with compact development, energy efficient mechanical systems, and on-site renewable power generation to address the array of health risks for building occupants associated with extreme heat events.

The results of the review demonstrate that an evidence base in the public health literature supports the application of green building projects pursuing LEED certification as a protective public health and environmental measure in locations confronting increased frequency and intensity of heat waves due to climate change. However, as Figure 1 outlines, it is not a direct causal relationship. The relationship between LEED requirements and benefits to public health and the built environment is mediated by environmental determinants of health; the type and degree of exposure; and, social/health, economic, and environmental outcomes. Furthermore, Table 2 displays a complex array of associations and potential pathways to enhance community resilience to extreme heat events. Whether or not a specific project will benefit from one or more of the strategies identified as leading to potential public health and/or built environment co-benefits will depend on its specific circumstances, and how the LEED effort fits into the project’s goals and the needs of the surrounding community. 

One of the major benefits of integrating public health considerations into the green building design process is increasing its emphasis on protecting vulnerable populations compared with current practice. In the case of extreme heat events, the most vulnerable groups include children [8,74,75,76,77,78,79,80,81,82], the elderly [8,74,75,77,78,79,80,81,82], populations suffering from chronic disease [8,74,75,77,78,79,80,81,82], families living in poverty [8,74,79,80,81,82,83,84], non-Hispanic Blacks [8,74,77,80,81,82,84,85,86], homeless populations [8,74,80,81,82,83,84,87], and outdoor workers [8,74,77,80,81,82,88,89], due to a combination of physiological, social, economic, and environmental factors. The systematic review returned results directly referencing vulnerable populations for nine of the twelve LEED credits under consideration. The common environmental determinant of health identified for Sustainable Sites Credit 5.1: Site Development—Protect or Restore Habitat, Sustainable Sites Credit 5.2: Site Development—Maximize Open Space, Sustainable Sites Credit 6.1: Stormwater Design—Quantity Control, Sustainable Sites Credit 6.2: Stormwater Design—Quality Control, and Sustainable Sites Credit 7.1: Heat Island Effect—Nonroof was “percentage vegetation in neighborhoods with vulnerable populations.” Similarly, the environmental determinant of health identified for Sustainable Sites Credit 7.2: Heat Island Effect–Roof was “exposure to high temperatures in urban areas,” a clear reference to lower income neighborhoods with a higher concentration of vulnerable families. The same environmental determinant of health was returned for all three credits in the Energy and Atmosphere category (Energy and Atmosphere Credit 1: Optimize Energy Performance, Energy and Atmosphere Credit 2: On-Site Renewable Energy, and Energy and Atmosphere Credit 3: Enhanced Commissioning) as wells as “power outage exacerbated by heat.” Again, lower income families are at higher risk than the general population of losing power, either for economic reasons or because they live in areas with substandard electrical infrastructure.

Clearly, architectural design decisions have larger ramifications on human and environmental health than are often acknowledged during the design process. By demonstrating the evidence base linking exposure pathways, environmental determinants of health, co-benefits to public health outcomes, and co-benefits to built environment outcomes, this review offers opportunities for collaboration among the public health, civil society, climate change, and green building sectors in a number of areas, including: health impact assessments, tool development, and social and health policy development.

### 4.1. Health Impact Assessments 

Health Impact Assessments (HIAs) deliver evidence-based recommendations designed to enhance the potential co-benefits and reduce the potential co-harms to health associated with a policy, project, or program [90]. Thanks in large part to the efforts of the Health Impact Project (http://www.pewtrusts.org/en/projects/health-impact-project), HIAs have become more common in the U.S. over the past fifteen years [90], influencing both climate change policies [91,92] and land use designs [90,93]. Future HIAs could use the evidence presented in this systematic review to recommend green building strategies as a method for linking desirable public health outcomes with built environment outcomes. For example, the systematic review found a strong association between ten LEED Credits and health co-benefits of mitigating the urban heat island (UHI) effect. Appropriately implemented, these green strategies can reduce the risk of exposure to extreme heat events and improve the thermal comfort of the site and neighborhood microclimate. UHI mitigation is also the most salient co-benefit of these strategies to the built environment, associated with all of the Credits in the Sustainable Sites category that were included in the review. An HIA could use this information to support a recommendation that UHI policies mandate or incentivize implementation of the LEED credits addressed in this review in neighborhoods that are highly vulnerable to extreme heat events. For example, the *35 Northampton Street Redevelopment Project Health Impact Assessment* performed by the Boston Health in All Policies Taskforce addresses climate change adaptation in the “Environmental Exposures” section of the HIA—alongside construction-related outdoor and indoor air quality, mid- to long-term indoor air quality, energy efficiency and climate change mitigation, and emergency preparedness and accessibility. The report recommends designing for passive cooling and installing Energy Star windows to protect building occupants from heat events [94]. A similar HIA in the future could use the systematic review presented in this article not only to provide more specific, evidence-based recommendations but also to develop a short-list of design strategies that would advance the goals of all of the sub-categories in the “Environmental Exposures” section of the report.

Design teams could use the results of this review as a basis for prioritizing LEED Credits that could reduce vulnerability to extreme heat events. Importantly, the systematic review continues beyond simply identifying LEED credits that can be used to minimize the negative health outcomes associated with the UHI effect. It also lays out the environmental determinants of health, which can be used to tailor specific design strategies to meet the intent of performance-based LEED credits. 

### 4.2. Tool Development 

This systematic review adds to the efforts underway by a number of green building tools to highlight the health benefits associated with their design recommendations. None of the comprehensive green building toolkits in the U.S.—such as LEED (http://www.usgbc.org/leed), EcoDistricts Protocol (http://ecodistricts.org/), Enterprise Green Communities (http://www.enterprisecommunity.com/ solutions-and-innovation/enterprise-green-communities/criteria), Living Building Challenge (http://living-future.org/lbc), and WELL Building Standard (http://www.wellcertified.com)—have identified which strategies in the current version of their standard could be used to both mitigate future GHG emissions and reduce the health effects of contemporary climate-related events. Many of them continue to treat climate change mitigation as unrelated to population health. The aspects of green building that have been highlighted as health promoting tend to be design elements encouraging physical activity, building materials reducing exposure to toxic chemicals, and increased ventilation. This systematic review expands that definition to protecting occupants and the surrounding community from the negative health effects of extreme heat. For example, all of the Sustainable Sites and Indoor Air Quality credits in the systematic review identify reducing vulnerability to heat stress and/or reducing the risk of heat-related morbidity and mortality as co-benefits to public health outcomes. The major health co-benefit associated with the Energy & Atmosphere credits is enhanced passive survivability—the ability of the building to reduce occupants’ exposure to heat, poor air quality, exertion, and psychological stress during a power outage [25].

This systematic review addresses links between a single green building tool (LEED) and the health effects of a single climatic event (extreme heat). Table A3 provides a crosswalk between the LEED credits covered in this review and credits in other leading sustainability best practice tools. However, additional systematic reviews must be performed to paint a complete picture of the level of protection from the negative health effects of climate change that could be achieved using current best practice guides. Additional research will be required to identify gaps that could be filled by future versions of these tools. Systematic reviews such as this one also serve the important role of demonstrating that green building tools in their current form can be used to enhance both climate change mitigation and adaptation, not just one or the other. 

### 4.3. Policy Development

This systematic review provides an evidence base for using green building strategies as a comprehensive approach to addressing climate change. Too often, separate policies are developed for climate change mitigation, climate change adaptation, zoning and land use, disaster planning, heat-related morbidity and mortality surveillance, emergency management, and sustainable development. Policy makers at all levels of government can use the risks of exposure and environmental determinants of health outlined in Table 2 to harmonize existing policies and craft future policies that take a more integrated approach to resolving environmental and public health challenges. For example, three environmental determinants of health were repeatedly called out in the systematic review: percentage vegetation in neighborhoods with vulnerable populations, exposure to high temperatures in vulnerable urban areas, and power outages exacerbated by heat. A typical policy response would treat these issues as separate policy initiatives. The parks department might spearhead a policy to increase vegetation. The public works and planning department might require white roofs on new construction to start reducing the urban heat island effect. The public utility might counter the risk of power outages by implementing rolling brownouts, instituting peak use surcharges, or looking for opportunities to increase capacity. And, the public health authorities might open emergency shelters for vulnerable populations, such as children [8]; the elderly [8,76,77,78,79,80]; homeless populations [87]; and, populations with pre-existing chronic health conditions such as cardiovascular, respiratory, and/or kidney disease [82], (p. 46). However, by considering these three environmental determinants of health within the context of extreme heat events, which are increasing in frequency and severity in many locations around the world, it is possible to develop a coordinated strategy that reduces the overall cost of intervention while simultaneously targeting the underlying cause — climate change. Public health surveillance systems, in particular, could use the information presented in this study to link heat-related morbidity and mortality surveillance with populations who are at high risk of negative health outcomes due to pre-existing chronic conditions. One population of particular concern are outdoor workers, including construction workers, who are at risk of extended exposure to high temperatures in the heat of the day unless accommodations to cool down are provided at the work site [80,88,89,95].

The evidence reviewed by this paper also indicates that built environment interventions should be tailored to address the environmental and social context specific to a location. A “one-size-fits-all” approach could lead to needless expenditures on the one hand and gaps in protective opportunities on the other. For example, the report *Assessing the Health Impacts of Urban Heat Island Reduction Strategies in the District of Columbia* [96] identifies the LEED rating system as a whole as a mechanism for reducing the UHI. Future policy background documents could use the results of this systematic review to identify the specific LEED credits most likely to achieve desired health benefits. From an international perspective, the *Lanet* Commission’s 2015 report on health and climate change [97] includes sustainable development as one of a number of “no regrets” mechanisms aimed at reducing the negative health effects of climate change. However, as this paper demonstrates, simply applying sustainable development principles in a given location will not necessarily lead to reducing social vulnerability or improving health outcomes in the face of climatic events. Sustainable development strategies should be tailored to address the underlying social and environmental determinants of health specific to the site and neighborhood where they will be implemented. 

### 4.4. Study Strengths and Limitations

This study filled a significant gap in the literature, identifying associations between specific green building strategies and the potential to reduce negative health outcomes of exposure to extreme heat events. The results speak to multiple disciplines (design, emergency management, public health, real estate, environmental consulting, etc.); because, the results are categorized by risk of exposure, environmental determinants of health, and co-benefits to both public health and the built environment. Because the study took a systematic approach to reviewing the entire LEED for New Construction rating system, the results can be used by a variety of disciplines to compare the health co-benefits of prioritizing one green building strategy over another one within the context of extreme heat events. 

However, the analysis included a number of limitations. First, and most importantly, a standard systematic review is not designed to map causal pathways or measure the relative strength of a single LEED Credit’s contribution to extreme heat resilience. Therefore, the results shared in this review simply identify associations between green building strategies and public health co-benefits without assessing the strength of association. A meta-analysis would strengthen this review’s results by addressing this limitation. However, it would have been difficult to perform for this project given, on the one hand, the variety of green building design strategies, environmental exposures, impacts on the built environment, and impacts on public health under review in comparison with, on the other hand, the limited number of studies included in the results measuring the strength of association between specific green building design strategies and the effect of heat events on buildings and building occupants. Additional research exploring the links between green building design and community resilience to extreme heat events has been published since 2012. As a result, a sufficient body of evidence may have become available to develop a quantitative comparison among some of the strategies identified in this article as either reducing exposure to extreme heat or enabling a building to continue to protect occupants during a heat event.

Secondly, the systematic review excluded all LEED Prerequisites, because they are required for all LEED certified projects. However, performing a systematic review of LEED Prerequisites would help identify the extent to which LEED certified projects in aggregate are enhancing resilience to the negative health effects of climatic events, regardless of which voluntary credits they pursue. The small number of peer-reviewed articles returned through the query process is another limitation for some LEED Credits under review. For example, the query for the parkland requirement under Sustainable Sites Credit 1: Site Selection generated only four citations, two of which were relevant. This limitation reflects the general need for further research linking architectural design and health outcomes, both in terms of climate change resilience and regarding other health concerns such as the built environment’s contribution to the prevalence of chronic disease. While a large body of research links land use decisions and transportation planning to public health outcomes [35,51,57,98,99,100,101,102,103,104,105,106,107,108,109,110], very little research investigates the role of building and site design on public health concerns—such as emergency preparedness and heat-related morbidity and mortality. This limitation might be partially overcome by increasing the number of years under review and/or by amplifying the PubMed results with an additional review targeting built environment journals and the grey literature. Additional systematic reviews are also necessary to expand the evidence base to other climate change-related events, such as hurricanes, drought, wildfire, and vector-borne disease.

Finally, the analysis would have been strengthened by incorporating credits from additional green building best practice standards, such as those outlined in Table A3. The final row in that table lists credits from those standards that might increase a building’s protective capacity during extreme heat events. In particular, future studies should research the potential protective impacts of: (1) developing a building adaptation plan as part of the design process, including extreme heat as one of the hazards under consideration; and, (2) explicitly encouraging on-site renewable power to be connected to the building in such a way that a portion of it would remain operational during a power outage.

## 5. Conclusions

Climate change poses a significant threat to public health. As a major contributor to both the cause of climate change and the ability for populations exposed to climatic events to adapt to changing conditions, the built environment represents an important component of efforts to enhance community resilience. While green building practices historically have prioritized climate change mitigation activities over adaptation, many of the strategies incorporated into best practice guidance documents such as LEED have the potential to reduce negative health outcomes following exposure to climatic events. This systematic review assessed the state of the evidence linking green building strategies in the LEED rating systems with the potential to reduce negative health outcomes following exposure to a significant climatic event: extreme heat.

The analysis found evidence that certain green building strategies have the potential to reduce the risk of negative health outcomes following exposure to heat. Key environmental determinants of health linking green building strategies and extreme heat events include: percentage vegetation (such as tree canopies) in neighborhoods with vulnerable populations and exposure to high temperatures in urban areas. Associated co-benefits to public health outcomes include: reducing vulnerability to heat stress and/or reducing the risk of heat-related morbidity and mortality. 

The results of this analysis, when coupled with a population health vulnerability assessment, offer opportunities for public health practitioners to collaborate with outside partners in three areas in particular: health impact assessments, tool development, and policy development. Future research should expand the evidence base linking building and land use design practices with climate change resilience and assess the strength of association between specific design practices and public health outcomes. Collaborations with civil society and schools of public health should involve relevant stakeholders in the training, applied research, and interventions required to prevent and reduce the impact of extreme heat in all populations, but specifically among vulnerable groups.

## Figures and Tables

**Table 1:** LEED Credits included in extreme heat resilience systematic review.

**LEED Credit Title** *Description*
**Sustainable Sites**
Sustainable Sites Credit 1: Site Selection *Avoid building on: prime farmland; land in 100-year flood plain; endangered species habitat; land within 100 feet of wetlands or 50 feet of water bodies; park land*.
Sustainable Sites Credit 2: Development Density and Community Connectivity *Locate project in a dense urban area or close to both a residential area and at least 10 basic services (i.e., grocery stores, etc.)*
Sustainable Sites Credit 5.1: Site Development—Protect or Restore Habitat *Limit disturbance of habitat on greenfield sites. Restore habitat on previously developed habitat*.
Sustainable Sites Credit 5.2: Site Development—Maximize Open Space *Increase vegetated open space*.
Sustainable Sites Credit 6.1: Stormwater Design—Quantity Control *Reduce the volume of stormwater that leaves the site after heavy precipitation events*.
Sustainable Sites Credit 6.2: Stormwater Design—Quality Control *Clean stormwater of total suspended solids*.
Sustainable Sites Credit 7.1: Heat Island Effect—Nonroof *Install light colored and pervious paving (i.e., roads, sidewalks, parking lots, etc) or place at least 1/2 of all parking spaces under cover*.
Sustainable Sites Credit 7.2: Heat Island Effect—Roof *Install light colored or vegetated roofs*.
**Water Efficiency**
None
**Energy and Atmosphere**
Energy and Atmosphere Credit 1: Optimize Energy Performance *Reduce energy use in the building*.
Energy and Atmosphere Credit 2: On-Site Renewable Energy *On-site installation of solar, wind, or other renewable energy source*.
Energy and Atmosphere Credit 3: Enhanced Commissioning *Perform commissioning (i.e., quality control) on all energy, domestic hot water, lighting, and renewable energy systems. Review building operations within 10 months after substantial completion of construction*.
**Materials and Resources**
None
**Indoor Environmental Quality**
Indoor Environmental Quality Credit 7.1: Thermal Comfort—Design *Design air conditioning (HVAC) systems and building envelope to meet standards for temperature, humidity, and airflow*.
**Innovation in Design**
None

Source: LEED Reference Guide for Green Building Design and Construction [22].

**Table 2:** Potential Co-Benefits (+) and Co-Harms (−) of LEED Credit Requirements on Heat-Related Public Health Outcomes, Categorized by Environmental Exposure.

LEED Credit Title *Description*	Target Public Health Interventions to Vulnerable Populations (+)	Risk of Heat-Related Morbidity and Mortality (+/−)	Risk of Cardiovascular Morbidity and Mortality (+/−)	Risk of Respiratory Disease (+/−)	Risk of Infectious Disease (+)	Risk of Vector-Borne Disease (+)	Risk of Under- and Mal-Nutrition (+)	Opportunity for Mental Health and Wellbeing (+)	Opportunity for Social Cohesion (+)
**Sustainable Sites**									
Sustainable Sites Credit 1: Site Selection *Avoid building on: prime farmland; land in 100-year flood plain; endangered species habitat; land within 100 feet of wetlands or 50 feet of water bodies; park land*.		L (+) S (+) U (+)		S (+)	S (+)		S (+)	L (+) S (+) U (+)	
Sustainable Sites Credit 2: Development Density and Community Connectivity *Locate project in a dense urban area or close to both a residential area and at least 10 basic services (i.e., grocery stores, etc.)*.		U (+/−)	U (+)	U (+)					U (+)
Sustainable Sites Credit 5.1: Site Development—Protect or Restore Habitat *Limit disturbance of habitat on greenfield sites. Restore habitat on previously developed habitat*.	U (+)	U (+)							
Sustainable Sites Credit 5.2: Site Development—Maximize Open Space *Increase vegetated open space*.	U (+)	U (+)							
Sustainable Sites Credit 6.1: Stormwater Design—Quantity Control *Reduce the volume of stormwater that leaves the site after heavy precipitation events*.	S (+) U (+)	S (+) U (+)		U (−)					
Sustainable Sites Credit 6.2: Stormwater Design—Quality Control *Clean stormwater of total suspended solids*.	S (+) U (+)	S (+) U (+)		U (−)					
Sustainable Sites Credit 7.1: Heat Island Effect—Nonroof *Install light colored and pervious paving (i.e., roads, sidewalks, parking lots, etc) or place at least 1/2 of all parking spaces under cover*.	U (+)	U (+)		U (−)					
Sustainable Sites Credit 7.2: Heat Island Effect—Roof *Install light colored or vegetated roofs*.	U (+)	U (+)							
**Energy and Atmosphere**									
Energy and Atmosphere Credit 1: Optimize Energy Performance *Reduce energy use in the building*.	P (+)	P (+) U (+)		P (+)		P (+)		P (+)	P (+)
Energy and Atmosphere Credit 2: On-Site Renewable Energy *On-site installation of solar, wind, or other renewable energy source*.	P (+)	P (+)		P (+)		P (+)		P (+)	P (+)
Energy and Atmosphere Credit 3: Enhanced Commissioning *Perform commissioning (i.e., quality control) on all energy, domestic hot water, lighting, and renewable energy systems. Review building operations within 10 months after substantial completion of construction*.	P (+)	P (+)		P (+)		P (+)		P (+)	P (+)
**Indoor Environmental Quality**									
Indoor Environmental Quality Credit 7.1: Thermal Comfort—Design *Design air conditioning (HVAC) systems and building envelope to meet standards for temperature, humidity, and airflow*.	P (+)	U (+)	U (+)	U (+)		P (+)		P (+)	P (+)

Notes: Environmental exposure notation: Land use changes increasing impervious cover (L); Power outage exacerbated by heat (P); Sprawl development (S); Urban heat island effect exacerbated by climate change (U).

**Table 3:** Potential Co-Benefits (+) and Co-Harms (−) of LEED Credit Requirements on Heat-Related Built Environment Outcomes, Categorized by Environmental Exposure.

LEED Credit Title *Description*	Urban Heat Island Effect/ Microclimate (+/−)	Air Pollution (+)	Density (+)	Focus Development in Areas with Services (+)	Access to Local, Productive Agricultural Land (+)	Pervious Surface, Shade (+)	Burden on Site Air Conditioning (+)	Burden on Electrical Grid (+)	Effectiveness of Passive Survivability (+)	Indoor Air Quality during Heat Events (+)
**Sustainable Sites**										
Sustainable Sites Credit 1: Site Selection *Avoid building on: prime farmland; land in 100-year flood plain; endangered species habitat; land within 100 feet of wetlands or 50 feet of water bodies; park land*.	S (+) U (+)	S (+)	S (+)	S (+)	S (+)	S (+) U (+)			L (+)	
Sustainable Sites Credit 2: Development Density and Community Connectivity *Locate project in a dense urban area or close to both a residential area and at least 10 basic services (i.e., grocery stores, etc.)*	U (+/−)			U (+)					U (+)	
Sustainable Sites Credit 5.1: Site Development—Protect or Restore Habitat *Limit disturbance of habitat on greenfield sites. Restore habitat on previously developed habitat*.	U (+)						U (+)			
Sustainable Sites Credit 5.2: Site Development—Maximize Open Space *Increase vegetated open space*.	U (+)						U (+)			
Sustainable Sites Credit 6.1: Stormwater Design—Quantity Control *Reduce the volume of stormwater that leaves the site after heavy precipitation events*.	S (+) U (+)	S (+)				S (+) U (+/−)	S (+) U (+)			
Sustainable Sites Credit 6.2: Stormwater Design—Quality Control *Clean stormwater of total suspended solids*.	S (+) U (+)	S (+)				S (+) U (+/−)	S (+) U (+)			
Sustainable Sites Credit 7.1: Heat Island Effect—Nonroof *Install light colored and pervious paving (i.e., roads, sidewalks, parking lots, etc) or place at least 1/2 of all parking spaces under cover*.	U (+)	U (+)				U (+)	U (+)			
Sustainable Sites Credit 7.2: Heat Island Effect—Roof *Install light colored or vegetated roofs*.	U (+)	U (+)				U (+)	U (+)			
**Energy and Atmosphere**										
Energy and Atmosphere Credit 1: Optimize Energy Performance *Reduce energy use in the building*.							P (+) U (+)	P (+) U (+)	P (+)	
Energy and Atmosphere Credit 2: On-Site Renewable Energy *On-site installation of solar, wind, or other renewable energy source*.								P (+)	P (+)	
Energy and Atmosphere Credit 3: Enhanced Commissioning *Perform commissioning (i.e., quality control) on all energy, domestic hot water, lighting, and renewable energy systems. Review building operations within 10 months after substantial completion of construction*.							P (+)	P (+)	P (+)	
**Indoor Environmental Quality**										
Indoor Environmental Quality Credit 7.1: Thermal Comfort—Design *Design air conditioning (HVAC) systems and building envelope to meet standards for temperature, humidity, and airflow*.	U (+)								P (+)	P (+) U (+)

Notes: Environmental exposure notation: Land use changes increasing impervious cover (L); Power outage exacerbated by heat (P); Sprawl development (S); Urban heat island effect exacerbated by climate change (U).

## Appendix A

**Table A1:** Association between LEED Credits and community resilience to extreme heat events: a review of the evidence.

LEED Credits	Requirements	MESH Query Terms	Relevant Citations ^a^	How Strategy Impacts Risk of Exposure	Environmental Determinants of Health	Co-Benefits to Public Health Outcomes	Co-Harms to Public Health Outcomes	Co-Benefits to Built Environment Outcomes	Co-Harms to Built Environment Outcomes
**Sustainable Sites Credit 1: Site Selection**	Avoid development in or adjacent to the following areas:								
	Prime farmland [18,37,38,39,40,41]	Agriculture Climate Change Facility Design and Construction Urbanization	6 (17)	Sprawl development Land use changes increasing impervious cover Urban heat island effect exacerbated by climate change	Air pollution Access to opportunities to exercise Dependence on automobiles Food and nutrition security Food safety Habitat fragmentation	Reduced risk of heat-related morbidity and mortality; cardiovascular morbidity and mortality; under- and mal-nutrition; infectious disease; interface between wildlife and humans.	None	Mitigated heat island effect. Reduced development in areas without services. Increased access to local, productive agricultural land.	None
	Endangered species habitat [42,43,44,45]	Biodiversity Urban Health	4 (7)	Sprawl development Urban heat island effect exacerbated by climate change	Air pollution Biodiversity in urban environments Disease-carrying vectors	Improved mental health and wellbeing. Reduced risk of respiratory disease. Reduced risk of malaria.	None	Mitigated heat island effect. Increased native vegetation and pervious surface. Increased street trees. Reduced ground-level ozone.	None
	Parkland [47,48]	Biodiversity Conservation of Natural Resources Facility Design and Construction	2 (4)	Sprawl development	Habitat fragmentation	Reduced risk of interface between wildlife and humans.	None	Mitigated heat island effect. Cluster development. Increase native vegetation and pervious surface.	None
	Requirements N/A to Heat: Floodplain Wetlands Water body								
**Sustainable Sites Credit 2: Development Density and Community Connectivity**	Locate building on an urban infill site or on a previously developed site that is located near a residential neighborhood and 10 basic services (such as grocery stores and banks). [32,33,34,35,36,49,50,51,52,53,54,55,56,57]	Climate Change Population Density Urban Health	14 (147)	Urban heat island effect exacerbated by climate change	Urban density Population density Street connectivity Access to multiple services Walkability	Community members available to check on socially isolated neighbors. Reduce risk of cardiovascular disease due to increased physical activity. Reduce heat stress via small-scale, targeted interventions.	Risk of increased risk factor for heat-related morbidity and mortality, cardiovascular disease, respiratory disease due to urban heat island.	Mitigated heat island effect. More likely to have access to transport. Small-scale improvements to infrastructure benefit a large number of people.	Risk of increased microclimate temperature.
**Sustainable Sites Credit 5.1: Site Development—Protect or Restore Habitat**	Limit habitat disturbance during construction or restore habitat. [3,58,59,60]	Climate Change Extreme Heat Environment Design	4 (15)	Urban heat island effect exacerbated by climate change	Percentage vegetation in neighborhoods with vulnerable populations.	Reduce vulnerability to heat stress. Target early heat warning system to neighborhoods with both high land surface temperature and vulnerable populations.	None	Improve thermal comfort of the site/ neighborhood microclimate. Reduce burden on air conditioning system.	None
**Sustainable Sites Credit 5.2: Site Development—Maximize Open Space**	Increase vegetated open space. [3,58,59,60]	Climate Change Extreme Heat Environment Design	4 (15)	Urban heat island effect exacerbated by climate change	Percentage vegetation in neighborhoods with vulnerable populations.	Reduce vulnerability to heat stress. Target early heat warning system to neighborhoods with both high land surface temperature and vulnerable populations.	None	Improve thermal comfort of the site/ neighborhood microclimate. Reduce burden on air conditioning system.	None
**Sustainable Sites Credit 6.1: Stormwater Design—Quantity Control**	Design the site to reduce the postdevelopment peak discharge quantity after heavy precipitation events. [3,41,58,59,60,61,62,63,64,65,66,67,68]	Climate Change Environment Design Extreme HeatUrbanization	12 (72)	Sprawl development Urban heat island effect exacerbated by climate change	Percentage vegetation in neighborhoods with vulnerable populations.	Reduce vulnerability to heat stress. Target early heat warning system to neighborhoods with both high land surface temperature and vulnerable populations.	Increasing pollen-producing plants could increase risk of respiratory disease.	Improve thermal comfort of the site/ neighborhood microclimate. Reduce burden on air conditioning system Reduce localized air pollution.	None
**Sustainable Sites Credit 6.2: Stormwater Design—Quality Control**	Design the site to remove pollution from stormwater runoff. [3,41,58,59,60,61,62,63,64,65,66,67,68]	Climate Change Environment Design Extreme Heat Urbanization	12 (72)	Sprawl development Urban heat island effect exacerbated by climate change	Percentage vegetation in neighborhoods with vulnerable populations.	Reduce vulnerability to heat stress. Target early heat warning system to neighborhoods with both high land surface temperature and vulnerable populations.	Increasing pollen-producing plants could increase risk of respiratory disease.	Improve thermal comfort of the site/ neighborhood microclimate. Reduce burden on air conditioning system Reduce localized air pollution.	None
**Sustainable Sites Credit 7.1: Heat Island Effect—Nonroof**	Shade impervious surfaces on-site or install light-colored or pervious surfaces. [3,41,58,59,60,61,62,63,64,65,66,67,68]	Climate Change Environment Design Extreme Heat Urbanization	13 (72)	Urban heat island effect exacerbated by climate change	Percentage vegetation in neighborhoods with vulnerable populations.	Reduce vulnerability to heat stress. Target early heat warning system to neighborhoods with both high land surface temperature and vulnerable populations.	Increasing pollen-producing plants could increase risk of respiratory disease.	Improve thermal comfort of the site/ neighborhood microclimate. Reduce burden on air conditioning system Reduce localized air pollution.	None
**Sustainable Sites Credit 7.2: Heat Island Effect—Roof**	Install light colored roof or vegetated roof. [3,41,62,63,64,65,66,67]	Climate Change Urbanization	8 (57)	Urban heat island effect exacerbated by climate change	Exposure to high temperatures in urban areas.	Reduce vulnerability to heat stress. Target early heat warning system to neighborhoods with both high land surface temperature and vulnerable populations.	None	Improve thermal comfort of the site/ neighborhood microclimate. Reduce burden on air conditioning system. Reduce localized air pollution. Preserve green space (including on the roof).	None
**Energy and Atmosphere Credit 1: Optimize Energy Performance**	Reduce energy use in building and/or increase use of on-site renewable power. [18,69,70,71,72,73,74]	Conservation of Energy Resources Cities Climate Change Disasters Electricity Environment Design Facility Design and Construction Urban Health	7 (19)	Urban heat island effect exacerbated by climate change Power outage exacerbated by heat	Exposure to high temperatures in urban areas. Power outage exacerbated by heat.	Reduced exposure to heat, poor air quality, exertion, and psychological stress during a power outage. Target public health interventions to vulnerable populations (i.e., elderly, patients dependent on electrically powered medical devices, etc.).	None	Increase the effectiveness of passive survivability. Reduce heat generated by buildings and increase thermal protection of occupants during events. Reduce burden on electrical grid. Reduce localized air pollution.	None
**Energy and Atmosphere Credit 2: On-Site Renewable Energy**	Use on-site renewable energy systems. [69,70,71,72,73,74]	Conservation of Energy Resources Cities Climate Change Disasters Electricity Urban Health	6 (14)	Power outage exacerbated by heat	Exposure to high temperatures in urban areas. Power outage exacerbated by heat.	Reduced exposure to heat, poor air quality, exertion, and psychological stress during a power outage. Target public health interventions to vulnerable populations (i.e., elderly, patients dependent on electrically powered medical devices, etc.).	None	Increase the effectiveness of passive survivability. Reduce heat generated by buildings and increase thermal protection of occupants during events. Reduce burden on electrical grid. Reduce localized air pollution.	None
**Energy and Atmosphere Credit 3: Enhanced Commissioning**	Verify that the building’s energy systems perform as efficiently as designed. [69,70,71,72,73,74]	Conservation of Energy Resources Cities Climate Change Disasters Electricity Urban Health	6 (14)	Power outage exacerbated by heat	Exposure to high temperatures in urban areas. Power outage exacerbated by heat.	Reduced exposure to heat, poor air quality, exertion, and psychological stress during a power outage. Target public health interventions to vulnerable populations (i.e., elderly, patients dependent on electrically powered medical devices, etc.).	None	Increase the effectiveness of passive survivability. Reduce heat generated by buildings and increase thermal protection of occupants during events. Reduce burden on electrical grid Reduce localized air pollution.	None
**Indoor Environmental Quality Credit 7.1: Thermal Comfort—Design**	Balance air temperature, humidity, and air speed to provide a space that is comfortable to occupants. [18,69,70,71,72]	Conservation of Energy Resources Cities Climate Change Disasters Electricity Environment Design Facility Design and Construction Urban Health	5 (16)	Urban heat island effect exacerbated by climate change Power outage exacerbated by heat	Exposure to high temperatures in urban areas. Power outage exacerbated by heat. Ventilation design.	Improve indoor air quality. Reduce risk of mold growth. Reduce risk of heat-related health effects; foodborne disease; potential for an increased rodent population. Reduced exposure to heat, poor air quality, exertion, and psychological stress during a power outage.	None	Increase the effectiveness of passive survivability. Increase indoor air quality protection of occupants during events.	None

Notes: ^a^ Total Queried in PubMed and Farr Report [30].

**Table A2:** MeSH Terms queried by LEED credit.

**LEED Credit Title** *Requirement(s): “MeSH Terms”*
Sustainable Sites Credit 1: Site Selection *Prime farmland: “Agriculture” AND “Facility Design and Construction” AND “Climate Change”;* *“Agriculture” AND “Urbanization” AND “Climate Change”*. *Land in 100-year flood plain: No queries relevant to heat*. *Endangered species habitat: “Biodiversity” AND “Urban Health”*. *Land within 100 feet of wetlands or 50 feet of water bodies: No queries relevant to heat*. *Park land: “Conservation of Natural Resources” AND “Biodiversity” AND “Facility Design and Construction”*.
Sustainable Sites Credit 2: Development Density and Community Connectivity *Locate project in a dense urban area: “Population Density” AND “Climate Change” AND “Urban Health”*. *Locate project close to both a residential area and at least 10 basic services (i.e., grocery stores, etc.):* *“Population Density” AND “Urban Health”*.
Sustainable Sites Credit 5.1: Site Development—Protect or Restore Habitat *Limit disturbance of habitat on greenfield sites: “Extreme Heat” AND “Environment Design”*. *Restore habitat on previously developed habitat: “Extreme Heat” AND “Climate Change”*.
Sustainable Sites Credit 5.2: Site Development—Maximize Open Space *Increase vegetated open space: “Extreme Heat” AND “Environment Design”; “Extreme Heat” AND “Climate Change”*.
Sustainable Sites Credit 6.1: Stormwater Design—Quantity Control *Reduce the volume of stormwater that leaves the site after heavy precipitation events:* *“Extreme Heat” AND “Environment Design”; “Extreme Heat” AND “Climate Change”;* *“Urbanization” AND “Climate Change”*.
Sustainable Sites Credit 6.2: Stormwater Design—Quality Control *Clean stormwater of total suspended solids: “Extreme Heat” AND “Environment Design”; “Extreme Heat” AND “Climate Change”; “Urbanization” AND “Climate Change”*.
Sustainable Sites Credit 7.1: Heat Island Effect—Nonroof *Install light colored and pervious paving (i.e., roads, sidewalks, parking lots, etc): “Extreme Heat” AND “Environment Design”; “Extreme Heat” AND “Climate Change”; “Urbanization” AND “Climate Change”*. *Place at least 1/2 of all parking spaces under cover: No queries relevant to heat*.
Sustainable Sites Credit 7.2: Heat Island Effect—Roof *Install light colored or vegetated roofs: “Urbanization” AND “Climate Change”*.
Energy and Atmosphere Credit 1: Optimize Energy Performance *Reduce energy use in the building: “Conservation of Energy Resources”[Mesh] AND “Climate Change” AND “Urban Health”; “Facility Design and Construction” AND “Environment Design” AND “Climate Change”;* *“Electricity” AND “Disasters” AND “Cities”*.
Energy and Atmosphere Credit 2: On-Site Renewable Energy *On-site installation of solar, wind, or other renewable energy source:* *“Conservation of Energy Resources” AND “Climate Change” AND “Urban Health”;* *“Electricity” AND “Disasters” AND “Cities”*.
Energy and Atmosphere Credit 3: Enhanced Commissioning *Perform commissioning (i.e., quality control) on all energy, domestic hot water, lighting, and renewable energy systems; review building operations within 10 months after substantial completion of construction:* *“Conservation of Energy Resources” AND “Climate Change” AND “Urban Health”;* *“Electricity” AND “Disasters” AND “Cities”*.
Indoor Environmental Quality Credit 7.1: Thermal Comfort—Design *Design air conditioning (HVAC) systems and building envelope to meet standards for temperature, humidity, and airflow: “Conservation of Energy Resources” AND “Climate Change” AND “Urban Health”;* *“Facility Design and Construction” AND “Environment Design” AND “Climate Change”;* *“Electricity” AND “Disasters” AND “Cities”*.

**Table A3:** Crosswalk of LEED Credits in Systematic Review and Other Green Building Best Practice Standards.

	BREEAM UK New Construction 2018 v2.0 [111]	CASBEE for Building (New Construction) 2014 [112]	Green Star Design & As Built v1.2 [113]	Living Building Challenge v3.1 [114]	2015 Enterprise Green Communities Criteria [115]	RELi Pilot v1.2.1 [116]	SITES v 2 [117]	Fitwel v1 [118]	WELL Building Standard v1 [119]
**Sustainable Sites**									
**Sustainable Sites Credit 1: Site Selection**	LE 02 Identifying and Understanding the Risks and Opportunities for the Project LE 03 Managing Negative Impacts on Ecology Pol 03 Flood and Surface Water Management	-	Land Use & Ecology 23 Ecological Value Land Use & Ecology 24 Sustainable Sites	Place 01 Limits to Growth	Location + Neighborhood Fabric 2. 1 Sensitive Site Protection	Risk Adaptation & Mitigation for Acute Events, HA R1 Sites of Avoidance & Repair Comprehensive Adaptation & Mitigation for a Resilient Present & Future PH PR2 Minimum Protection for Prime Habitat & Floodplain Functions Comprehensive Adaptation & Mitigation for a Resilient Present & Future PH Pc5 Ecological PHD: Protect Wetlands & Avoid Slopes and Adverse Geology	Site Context P1.1 Limit Development on Farmland Site Context P1.3 Conserve Aquatic Ecosystems Context P1.4 Conserve Habitats for Threatened and Endangered Species	-	-
**Sustainable Sites Credit 2: Development Density and Community Connectivity**	Tra 02 Sustainable Transport Measures	Q.3.3. Outdoor Environment (On-Site): Local Characteristics & Outdoor Amenity	Land Use & Ecology 24 Sustainable Sites	Place 01 Limits to Growth	Location + Neighborhood Fabric 2.2 Connections to Existing Development & Infrastructure Location + Neighborhood Fabric 2.3-2.4 Compact Development Location + Neighborhood Fabric 2.5 Proximity to Services	Comprehensive Adaptation & Mitigation for a Resilient Present & Future CV Pc3 Community Connectivity: Mixed-Use Commercial, Housing & Public/Community Space	Site Context c1.6 Locate Projects Within Existing Developed Areas	Entrances & Ground Floor 4.5 Provide at least one Publicly Accessible Use on the Ground Floor to Encourage Pedestrian Activity Entrances & Ground Floor 4.6 Provide a Dedicated Display Advertising Amenities within Walking Distance of the Building	Fitness 67 Exterior Active Design
**Sustainable Sites Credit 5.1: Site Development—Protect or Restore Habitat**	Le 01 Site Selection LE 03 Managing Negative Impacts on Ecology LE 04 Change and Enhancement of Ecological Value	Q.3.1. Outdoor Environment (On-Site): Preservation & Creation of Biotope	Land Use & Ecology 24 Sustainable Sites	Place 01 Limits to Growth	Location + Neighborhood Fabric 2.6-2.7 Preservation of and Access to Open Space	Risk Adaptation & Mitigation for Acute Events HA Pc6 Provide Environmental Protection & Remediation for Parks & Preserves	Site Context P1.4 Conserve Habitats for Threatened and Endangered Species	-	-
**Sustainable Sites Credit 5.2: Site Development—Maximize Open Space**	Hea 07 Safe and Healthy Surroundings	Q.3.1. Outdoor Environment (On-Site): Preservation & Creation of Biotope	Land Use & Ecology 23 Ecological Value	Place 01 Limits to Growth	Location + Neighborhood Fabric 2.6-2.7 Preservation of and Access to Open Space	-	Site Contect c1.6 Locate Projects Within Existing Developed Areas	Outdoor Spaces 3.1 Provide an Outdoor Space Amenity that is Accessible from a Building Entrance Outdoor Spaces 3.4 Provide a Healing Garden or Therapeutic Landscape Amenity	Mind 88 Biophilia I, Qualitative Mind 100 Biophilia II, Quantitative
**Sustainable Sites Credit 6.1: Stormwater Design—Quantity Control**	Pol 03 Flood and Surface Water Management	-	Emissions 26 Storm Water	Water 05 Net Positive Water	Site Improvements 3.6 Surface Stormwater Management	Risk Adaptation & Mitigation for Acute Events HA Pc2 Adaptive Design for Extreme Rain, Sea Rise, Storm Surge & Extreme Weather, Events & Hazards Comprehensive Adaptation & Mitigation for a Resilient Present & Future EW PR1 Minimum Water Efficiency & Resilient Water and Landscapes Comprehensive Adaptation & Mitigation for a Resilient Present & Future EW Pc1 Plan for Rainwater Harvesting, Resilient Landscapes & Food Production	Water P3.1 Manage Precipitation On Site Water c3.3 Manage Precipitation Beyond Baseline-95^th^ Percentile Event	-	-
**Sustainable Sites Credit 6.2: Stormwater Design—Quality Control**	Pol 03 Flood and Surface Water Management	-	Emissions 26 Storm Water	Water 05 Net Positive Water	Site Improvements 3.6 Surface Stormwater Management	Risk Adaptation & Mitigation for Acute Events HA Pc2 Adaptive Design for Extreme Rain, Sea Rise, Storm Surge & Extreme Weather, Events & Hazards Comprehensive Adaptation & Mitigation for a Resilient Present & Future EW PR1 Minimum Water Efficiency & Resilient Water and Landscapes Comprehensive Adaptation & Mitigation for a Resilient Present & Future EW Pc1 Plan for Rainwater Harvesting, Resilient Landscapes & Food Production	Water P3.1 Manage Precipitation On Site Water c3.3 Manage Precipitation Beyond Baseline-95^th^ Percentile Event	-	-
**Sustainable Sites Credit 7.1: Heat Island Effect—Nonroof**	-	Q.3.3.2 Outdoor Environment (On-Site): Local Characteristics & Outdoor Activity, Improvements of the Thermal Environment on Site LR.3.2.2. Off-Site Environment: Consideration of Local Environment, Heat Island Effect	Land Use & Ecology 25 Heat Island Effect	Place 01 Limits to Growth	Site Improvements 3.7 Reducing Heat-Island Effect: Paving	Comprehensive Adaptation & Mitigation for a Resilient Present & Future EW Pc6 Reduced Site Environmental Impacts, Lighting, Heat-Island, Airborne Toxins	Soil + Vegetation c4.9 Reduce Urban Heat Island Effects Soil + Vegetation c4.10 Use Vegetation to Minimize Building Energy Use	-	-
**Sustainable Sites Credit 7.2: Heat Island Effect—Roof**	-	Q.3.3.2 Outdoor Environment (On-Site): Local Characteristics & Outdoor Activity, Improvements of the Thermal Environment on Site LR.1.1. Energy: Control of Heat Load on the Outer Surface of Buildings LR.3.2.2. Off-Site Environment: Consideration of Local Environment, Heat Island Effect	Land Use & Ecology 25 Heat Island Effect	Place 01 Limits to Growth	Materials 6.11 Reduced Heat-Island Effect, Roofing	Risk Adaptation & Mitigation for Acute Events HA Pc4 Passive Thermal Safety, Thermal Comfort & Lighting Design Strategies Comprehensive Adaptation & Mitigation for a Resilient Present & Future EW Pc2 Plan the Site and Orientation for Sun & Wind Harvesting, Natural Cooling	-	-	-
**Energy and Atmosphere**									
**Energy and Atmosphere Credit 1: Optimize Energy Performance**	Ene 01 Reduction of Energy Use and Carbon Emissions Ene 04 Low Carbon Design	LR.1.2. Energy: Natural Energy Utilization LR.1.3. Energy: Efficiency in Building Service System LR.3.2.3. Off-Site Environment: Consideration of Local Environment, Load on Local Infrastructure	Energy 15 Greenhouse Gas Emissions Energy 16 Peak Electricity Demand Reduction	Energy 06 Net Positive Energy	Location + Neighborhood Fabric 2.10 Passive Solar Heating/Cooling Energy Efficiency 5.1 Building Performance Standard Energy Efficiency 5.2 Additional Reductions in Energy Use, Nearing Net Zero	Comprehensive Adaptation & Mitigation for a Resilient Present & Future EW Pr2 Minimum Energy Efficiency & Atmospheric Impacts Comprehensive Adaptation & Mitigation for a Resilient Present & Future EW Pc4 Energy Optimization	Soil + Vegetation c4.10 Use Vegetation to Minimize Building Energy Use Operations + Maintenance c8.5 Reduce Outdoor Energy Consumption	-	Air 19 Operable Windows
**Energy and Atmosphere Credit 2: On-Site Renewable Energy**	Ene 01 Reduction of Energy Use and Carbon Emissions Ene 04 Low Carbon Design Wst 05 Adaptation to Climate Change	LR.1.3. Energy: Efficiency in Building Service System	Energy 15 Greenhouse Gas Emissions Energy 16 Peak Electricity Demand Reduction	Energy 06 Net Positive Energy	Energy Efficiency 5.7 Photovoltaic / Solar Hot Water Ready, Renewable Energy	Comprehensive Adaptation & Mitigation for a Resilient Present & Future EW Pc4 Energy Optimization	Operations + Maintenance c8.6 Use Renewable Sources for Landscape Electricity Needs	-	-
**Energy and Atmosphere Credit 3: Enhanced Commissioning**	Man 04 Commissioning and Handover Man 05 Aftercare	-	Management: 2 Commissioning & Tuning Management 4 Building Information	Energy 06 Net Positive Energy	Energy Efficiency 5.1 Building Performance Standard Operations, Maintenance + Resident Engagement 8.1 Building O&M Manual & Plan	Panoramic Approach PR 3 Commissioning & Long-Term Monitoring / Maintenance	-	-	A03.2 Conduct System Balancing A09.1 Design Healthy Envelope and Entryways
**Indoor Environmental Quality**									
**Indoor Environmental Quality Credit 7.1: Thermal Comfort—Design**	Hea 04 Thermal Comfort	Q1.2. Indoor Environment: Thermal Comfort	Indoor Environmental Quality 14 Thermal Comfort	Health & Happiness 07 Civilized Environment Imperative	-	Risk Adaptation & Mitigation for Acute Events HA Pc4 Passive Thermal Safety, Thermal Comfort & Lighting Design Strategies	-	-	Air 19 Operable Windows Comfort 76 Thermal Comfort
**Additional green building strategies not included in the systematic review that could increase a building’s protective capacity during extreme heat events**	Ene 08 Energy Efficient Equipment Wst 05 Adaptation to Climate Change	Q.2.2.4. Quality of Service: Durability & Reliability, Reliability LR.3.2.1. Off-Site Environment: Consideration of Local Environment, Air Pollution	Management 3 Adaptation & Resilience	-	Integrative Design 1.2 Resident Health and Well-Being Integrative Design 1.3 Resilient Communities Energy Efficiency 5.4 ENERGY STAR Appliances Energy Efficiency 5.8b Resilient Energy Systems, Islandable Power	Panoramic Approach R1 Study: Project Short-Term Hazard Mitigation and Adaptation Needs Including Climate Panoramic Approach c2 Establish a Sustainability & Resiliency Management System Risk Adaptation & Mitigation for Acute Events Hazard Preparedness Risk Adaptation & Mitigation for Acute Events HA Pc3 Advanced Emergency Operations	-	-	-
